# Evaluating a theory-based intervention for improving eHealth literacy in older adults: a single group, pretest–posttest design

**DOI:** 10.1186/s12877-022-03545-y

**Published:** 2022-11-29

**Authors:** Sun Ju Chang, Kyoung-eun Lee, Eunjin Yang, Hyunju Ryu

**Affiliations:** 1 College of Nursing & Research Institute of Nursing Science, Seoul National University, Seoul, Republic of Korea; 2 Department of Nursing, Sunmoon University, Asan, Republic of Korea; 3 Mo-Im Kim Nursing Research Institute, Yonsei University College of Nursing, Seoul, Republic of Korea; 4 College of Nursing, Seoul National University, Seoul, Republic of Korea

**Keywords:** Older adults, IMB model, Intervention mapping, eHealth, eHealth literacy, Intervention

## Abstract

**Background:**

The Internet is considered an important channel for providing health information to older adults. We developed an intervention to improve eHealth literacy in older adults according to the information-motivation-behavioral skills (IMB) theory and Intervention Mapping. This study aimed to analyze the effect of a developed intervention on information, motivation, behavioral skills, and behaviors related to eHealth information in older adults.

**Methods:**

Forty-six older adults over the age of 65 were recruited from two senior welfare centers in a city in South Korea. We divided the participants into four groups and conducted one intervention per group from March to December 2019. One intervention consisted of 5 sessions and was performed once a week (2 h/1 time) for 5 weeks, culminating in a total lecture time of 10 h. One lecture instructor and two assistant instructors supported the participants in the computer practices.

**Results:**

Participants’ computer/web knowledge, perceived ease of use, perceived enjoyment, and attitude toward eHealth information showed statistically significant increases. The eHealth literacy efficacy score, searching performance score, and understanding score were also significantly increased. However, there was no significant difference in perceived usefulness.

**Conclusion:**

The application of the current theory-based methodology can improve the quality of research in eHealth literacy. Additionally, various interventions should be developed and continuously applied to improve eHealth literacy among older adults.

## Introduction

Information and communication technologies (ICT), represented by the Internet, have been deeply embedded in our daily lives. Since the concept of ubiquitous computing was first introduced in 1991, many infrastructures related to the systems have been equipped to enable people to readily access the Internet [1]. Consequently, more than half of the world’s population were using the Internet by 2019, which has more than doubled since the past decade [2]. The Internet usage rate of older adults, who were considered underprivileged in the digital world, has been steadily increasing over time. Approximately 75% of those aged 65 years or older in the United States and 89.1% of people in their 60s in South Korea, use the Internet [3, 4]. On the Internet, older adults get news and information, communicate with others, enjoy leisure activities, and shop for groceries [4]. In particular, the Internet has been regarded as an attractive and important source of health information for older adults, who are the largest consumers of healthcare services [5].

Previous studies have reported that a considerable number of older adults use the Internet to access health-related information [6–8], and that they focus on the positive functions of health information on the Internet [9]. However, other studies have found that the majority of older adults are still reluctant to use the Internet to search for health information [10, 11]. eHealth information access by older adults is speculated to be linked with challenges such as technical difficulties, lack of learning opportunities, lower eHealth literacy, and concerns about the quality of eHealth information [12–14]. Some researchers have developed intervention programs to improve eHealth literacy by reflecting older adults’ characteristics and needs, and have reported significant changes in knowledge and skills regarding eHealth information [15–17]. However, the findings from a systematic review that analyzed studies targeting eHealth literacy in older adults showed that less than half of the intervention programs were developed based on theory or theoretical frameworks, which is essential for achieving better results as it provides comprehensive explanations about complex nursing phenomena [18, 19]. Even studies using theories or theoretical frameworks did not describe the transparent program development processes, such as how the theory’s concept has been translated and incorporated into the programs [18]. As such, studies that are not based on theories or based on inadequate theories could not comprehensively explain eHealth literacy in older adults [18]. Another systematic review suggested the application of theory to improve the quality of research on eHealth literacy interventions [20]. In addition, factors such as credibility and computer skills, which are known as imperative components related to using eHealth information in older populations, should be comprehensively reflected in intervention programs [18, 20].

Therefore, we developed and preliminarily tested an information-motivation-behavioral skills (IMB) model-based intervention to improve eHealth literacy in older adults [21], and finalized the intervention program. The IMB model is a behavior-oriented theory that is widely used in the development of health promotion programs to change health behavior [22]. The IMB model consists of four domains: information, motivation, behavioral skills, and behavior, positing that information, motivation, and behavioral skills directly and indirectly influence health behavior [23]. This study aims to examine the program’s effects on information, motivation, behavioral skills, and behaviors, related to eHealth information among older adults. The specific hypotheses, based on the IMB model, are as follows:

1. Information domain: Intervention will increase computer/web knowledge in older adults.
2. Motivation domain: Intervention will change the perceived usefulness, perceived ease of use, perceived enjoyment, and attitude toward Internet health information in older adults.
3. Behavioral skills domain: Intervention will increase eHealth literacy in older adults.
4. Behavior domain: Intervention will improve search performance, the level of understanding of eHealth information, and health behavior decision-making in older adults.

## Methods

### Study Design

This quasi-experimental study uses a one-group, pretest-posttest design to evaluate the intervention’s effects on information, motivation, behavioral skills, and behaviors related to eHealth information among older adults.

### Participants

Convenience sampling was used to recruit older adults aged above 65 years from two senior welfare centers in South Korea. Among the subjects who could communicate, understand the study’s purpose, and respond with deliberate voluntary participation, we selected those who met the following criteria: (1) age ≥ 65 years and, (2) no cognitive impairments to hinder participation in the study (i.e., Korean Mini-Mental Status Examination score > 24). The researchers provided sufficient information about the study’s purpose and method to the selected participants through face-to-face interviews; all participants provided written informed consent.

The G * power 3.1 program was used to calculate the number of participants required for the study. Based on a previous study [24], the effect size was set to 0.5. Also, we assigned a significance level of 0.05 and a power of 0.95 to calculate the sample size using a single group means comparison; the result was 45. We tried to recruit 60 participants considering that the dropout rate is 20% [25], but only 50 participants were recruited. We divided the 50 participants into four groups. Considering that this program was organized to combine computer practice and lectures, we determined that fewer than 15 member interventions were appropriate [26]. For each group, one intervention consisting of 5 sessions was performed. The number of participants in each group was as follows: G1(Group 1) intervention: 13; G2 intervention: 11; G3 intervention: 14; G4 intervention: 12. Four out of 50 participants failed to complete the program or failed to respond to the questionnaire; thus, 46 participants were finally selected. Post-hoc power analysis was conducted using G*power 3.1 again, and the following specifications: effect size = 0.8, significance level = 0.05, total sample size = 46. As a result, the power was over 0.955, indicating that the sample size (i.e., 46) was sufficient for the present analysis.

### Intervention program

This intervention was developed based on the IMB model [23] and Intervention Mapping guidelines [27]. The detailed development process and the pilot study’s results were also found in a previous study [21].

This intervention was conducted over a total of 10 h, once a week (2 h each), for five weeks. The instructors were researchers participating in the development of this intervention, where one main instructor was dedicated to teaching, and two assistant instructors assisted the participants’ computer training. The ratio of participants to instructors was 1:3–5 throughout the study.

This intervention was divided into five sessions. Each session’s main contents and the websites’ characteristics are listed in Table 1. Each session consisted of an introduction (20 min), a main session (65 min), and a summary (15 min). In the introduction, the instructor briefly outlined the intervention process, introduced research team members, and presented the learning goals. The main session was conducted through lectures and computer exercises. In summary, the instructors and participants checked the goals achieved and facilitated the quiz. With the help of assistant instructors, the participants were able to participate in the intervention actively and dynamically. Furthermore, we scripted the intervention protocol in detail for its widespread use.

**Table 1:** Contents of the intervention for improving eHealth literacy for older adults

Intervention Contents	Characteristics of Websites	Teaching Chanel/Effect
Session 1. Basic computer knowledge Basic computer and web-related knowledge Using the computer mouse Basic internet terms		PPT lecture/ Attention Video demonstration/ Selective Perception and attention Computer practice/ Learning reinforcement Discussion, Quiz/ Information reproduction and reinforcement Handout/ Information Reinforcement
Session 2. Use and understanding of the National Health Information Portal (NHIP) Accessing the NHIP Searching for health information in the NHIP Using the scroll bar for searching information Playing a video in the NHIP	The NHIP https://health.kdca.go.kr/ Website created by the Korea Disease Control and Prevention Agency. Providing reliable and high-quality health information. Providing integrated and linked information related to medical institutions, medicines, and health insurance.	
Session 3. Utilization of the National Health Information Portal (NHIP) Downloading data from the data room of the NHIP Searching for disease symptoms on the NHIP Searching for drug/food information on the NHIP Navigating other public institutions websites through the NHIP		
Session 4. Use of Korean search engine “Naver” Accessing Naver Entering health information search words Exploring the Knowledge encyclopedia on Naver Searching for reliable health information on Naver	Naver https://www.naver.com Korea’s most representative and largest internet search engine. Providing community services such as blogs, cafes, posts, and Knowledge encyclopedia, including portal services.	
Session 5. Evaluating the reliability of health information websites Finding the sponsor of health information websites Checking the purpose of the website Checking the information of authors Checking the latest updated date of information Finding the privacy policy of the websites Evaluating reliability of the website	Health-In https://www.nhis.or.kr Website operated by the Korean National Health Insurance Corporation. Providing reliable information for health screenings and health insurance.	

This intervention was conducted four times for four groups of 46 participants in two senior welfare centers with computer labs for practical computer training. In addition, the effectiveness of the intervention was evaluated thrice: before the intervention (baseline), immediately after the intervention (post 1), and two months after the intervention (post 2).

### Measurements

#### Demographic and internet-related characteristics

Participants were asked about their demographic characteristics, including age, sex, educational level, marital status, economic status, and comorbidities, in addition to their eHealth information search experience, types of health information searched on the Internet, the duration and frequency of Internet use, and types of Internet search tools.

#### Program satisfaction

Satisfaction with the program was measured using five questions used in a study by Xie [28]. This scale consists of questions about evaluating the level of lectures, the usefulness and satisfaction of the program, the intention to re-engage in the program, and whether or not to recommend to others. Each item is configured to respond from 1 (very bad) to 5 (very good), and the total score is within the range of 5–25. The alpha coefficient for this scale was 0.759.

#### Information: computer/web knowledge

For computer and web knowledge, a scale developed by Xie was used [28]. This scale consists of 19 questions, including asking for web-related terms and writing down the names of the computer and website images (e.g., mouse, keyboard, link, scroll bar) after viewing these images. The alpha coefficient for this scale was 0.814.

#### Motivation: perceived usefulness, perceived ease of use, and perceived enjoyment of internet information technology

In 2013, the technology Acceptance Model 3 (TAM 3) was revised to a Korean version [29, 30]. Among the modified TAM 3 scales, we selected four, four, and three items about perceived usefulness, perceived ease of use, and perceived enjoyment of eHealth information, respectively. Each item is scored on a 7-point Likert scale, and the total score is calculated by summing the individual items’ scores in each area. The higher the score in each area, the higher the perceived usefulness, perceived ease of use, and perceived enjoyment. While the previous study’s alpha coefficient of each area was higher than 0.7 [29], in this study, 0.927 was adopted for perceived usefulness, 0.822 for perceived ease of use, and 0.928 for perceived enjoyment.

#### Motivation: attitudes toward eHealth information

Attitudes toward eHealth information were measured using a scale developed by Jung, Kang, Suk, & Kim [31]. This scale consists of five items rated on a 5-point Likert scale, examining an individual’s thoughts on eHealth information in terms of reliability, usefulness, specificity, accuracy, and updatedness. Responses were recorded in the range of 1 (I do not agree very much) to 5 (I agree very much). The higher the total sum of the scores of each item, the more positive the attitude toward eHealth information. While the Cronbach’s alpha was 0.80 in Jung Kang, Suk, & Kim’s study [31], it was 0.819 in this study.

#### Behavioral skills: eHealth literacy efficacy

eHealth literacy efficacy is an individual’s perception and confidence in their ability to search for and understand eHealth information [21, 32]. The eHealth Literacy Scale (eHEALS) was developed by Norman and Skinner based on self-efficacy theory [33]. It was translated into Korean through a cultural revising process called K-eHEALS [34]. In this study, the K-eHEALS was used to assess the eHealth literacy efficacy. The K-eHEALS consists of 10 items scored on a 5-point Likert scale. The response of each item ranged from 1 (I do not agree very much) to 5 (I agree very much). In addition, only eight items (3 to 10) were calculated for the total score. The total score was calculated from 8 to 40, where the higher the total score, the higher the eHealth literacy. While Cronbach’s alpha in the previous study was 0.88 [33], it was 0.903 in this study.

#### Behaviors: searching for eHealth information

The eHealth information-seeking behavior was assessed in terms of accuracy and time (the lesser the time taken, the higher the efficiency), which is a modification of Sharit et al.’s measurement method for searching task performance [35]. We presented one topic to the participants (baseline for vaccinations for older adults, post 1 for stroke, post 2 for angina), and then recorded the time taken by participants to access the Internet and enter the National Health Information Portal (https://health.cdc.go.kr/) to find the relevant topic. A research assistant tracked the time from the click of the Internet button to accessing the relevant web page using a stopwatch; two points were provided if the searched web page was correct, and 0 if it was incorrect. The better the search, the higher the search score [35].

#### Behaviors: understanding eHealth information

To analyze the degree of understanding information, participants’ understanding of the search topic was assessed. Each topic was composed of 10 questions; the correct answer was scored 1, and the incorrect answer was scored 0. Thus, the higher the score, the higher the understanding of the topic. The questions were validated by two nursing professors.

#### Behaviors: health behavior and decision-making changes

The scale developed by Xie [24] was based on questions from the Kaiser Family Foundation survey [36] using the Pew Internet study [37]. This scale consists of 12 items. In this study, the Internet site was chosen to suit the Korean context, and it consisted of nine items. The revised items were applied to the participants through content validity by two nursing professors. The Cronbach’s alpha for this scale was. 803.

### Data collection

The intervention and data collection for this study was conducted from March to December 2019. Firstly, to recruit the participants, we contacted senior welfare centers equipped with a computer room, explained the study’s purpose, and requested their cooperation. Subsequently, data collection and intervention were conducted in two locations. The self-report questionnaire consisted of 69 questions, and performance evaluation (Internet searching) was conducted by accessing the Internet to find a specific topic. The time required to complete the questionnaire and evaluate performance (Internet search) was approximately 40 min.

### Ethical considerations

This study was conducted after obtaining approval from the institutional review board (IRB) of the university to which the researchers were affiliated (IRB No. 1901/002–011). Before the study commenced, we explained the study’s purpose and procedures to the participants and provided sufficient information on the protection of human rights and the guarantee of data anonymity. All participants who completed the questionnaire were given a gift card.

### Data analysis

The collected data were statistically analyzed using SPSS version 23.0. (IBM, New York, United States). Firstly, demographic, and Internet-related characteristics of the participants were examined by computing descriptive statistics. Secondly, the effectiveness of the intervention at three points (baseline, post 1, and post 2) was analyzed using a generalized estimating equation (GEE). In addition, a useful statistical technique was required to minimize the effect of missing values in repeated measurements. GEE has been suggested as an effective method for analyzing longitudinal data of the same subject measured at different time points [38].

## Results

### Demographic and internet-related characteristics

Tables 2 and 3 present the participants’ demographic and Internet-related characteristics. The 46 participants included 24 men (52.2%) and 22 women (47.8%). Their mean age was 75.52 years (SD = 5.50). Approximately 61% of the participants had experience of using the Internet, and approximately 80% had comorbidities. Regarding their educational level, 18 older adults with a bachelor’s degree had the highest frequency (39.2%). In addition, 30 (65.2%) participants answered that they had no economic difficulties.

**Table 2:** Demographic characteristics of the participants (N = 46)

Variable	n (%)	Mean (SD)
Gender		
Male	24 (52.2)	
Female	22 (47.8)	
Age		75.52 (5.50)
eHealth searching experience		
Yes	28 (60.9)	
No	18 (39.1)	
Marital status		
Married	33 (71.7)	
Single	13 (28.3)	
Comorbidity		
Yes	37 (80.4)	
No	9 (19.6)	
Educational level		
Elementary school	3 (6.5)	
Middle school	7 (15.2)	
High school	12 (26.1)	
≥Diploma	6 (13.0)	
≥Bachelor’s	18 (39.2)	
Economic status		
Very difficult for daily living	3 (6.5)	
Somewhat difficult for daily living	13 (28.3)	
No difficult for daily living	30 (65.2)	

**Table 3:** Internet use-related characteristics of the participants (N = 46)

Variable		n (%)	Mean (SD)
Types of health information searched on the Internet			
Disease		24 (52.2)	
Method of treatment		17 (37.0)	
Drugs		13 (28.3)	
Diagnosis Method		10 (21.7)	
Health behaviors		12 (26.1)	
None		9 (19.6)	
Others		4 (8.7)	
Internet usage period			
Less than 1 year		10 (21.7)	
1 year to 5 years		11 (23.9)	
More than 5 years		17 (37.0)	
Never tried		8 (17.4)	
Internet usage time during the day			
Less than 1 h		17 (37.0)	
1 to 3 h		16 (34.8)	
Rarely		13 (28.2)	
Types of Internet Search Tools			
Computer		32 (69.6)	
Mobile Phone (Smart Phone)		25 (54.3)	
Tablet PC		4 (8.7)	
Frequently searched web-page types			
Social Network Service (SNS)		14 (30.4)	
Blog		11 (23.9)	
Café		4 (8.7)	
News		9 (19.6)	
Sites of specific institutions (hospitals, etc.)		11 (23.9)	
Program satisfaction			22.28 (2.38)

Multiple answers were available for the types of Health Information searched on the Internet, the types of Internet Search Tools and frequently searched web-page types

A total of 24 participants searched the Internet for health information related to diseases (52.2%), and 17 (37.0%) searched for information related to treatment for diseases. Regarding the period of Internet use, 17 (37.0%) participants were Internet users for over five years, while 10 (21.7%) were Internet users for less than one year, indicating that intermediate users and beginners for Internet use coexisted. A total of 17 (37.0%) participants answered that they used the internet for less than an hour during the day. Thirty-two (69.6%) used a computer the most as an internet search tool. Regarding the types of webpages that were mainly viewed, 14 (30.4%) used SNS, and 11 (23.9%) searched for a specific homepage or blog, such as a hospital homepage. This eHealth information program’s average satisfaction level was 22.28 (SD = 2.38) (range: 5–25).

### Effects of a theory-based intervention for improving ehealth literacy in older adults

For the distribution of all variables, the identity link function of linear distribution was used, and GEE was performed by designating an autoregressive (AR) (1) model as the correlation matrix between repeated measured variables. Table 4 presents the results verified by GEE.

**Table 4:** Effects of the intervention for improving eHealth literacy for the elderly (N = 46)

Constructs of IMB model and Variables	Baseline	Post 1	Post 2	χ^2^	*P*	*Cohen’s d*
	**estimated Mean ± SE**	**estimated Mean ± SE**	**estimated Mean ± SE**			
Information						
Computer/web knowledge	8.47 ± 38.23	14.06 ± 38.38	12.94 ± 38.40	60.04	< 0.001	0.57
Motivation						
Perceived usefulness	22.81 ± 37.07	24.61 ± 37.19	23.95 ± 36.91	5.91	0.052	
Perceived ease of use	20.12 ± 31.64	22.88 ± 31.63	22.49 ± 31.55	17.86	< 0.001	0.88
Perceived enjoyment	15.66 ± 22.31	18.16 ± 22.34	17.41 ± 22.27	23.06	< 0.001	0.87
Attitudes toward eHealth Information	17.05 ± 20.71	19.24 ± 20.79	18.83 ± 20.65	18.47	< 0.001	0.52
Behavior skills						
eHealth literacy	27.52 ± 36.48	32.67 ± 36.28	31.38 ± 36.38	72.76	< 0.001	0.45
Behaviors						
Searching for eHealth Information	1.56 ± 49.55	23.06 ± 49.02	9.65 ± 49.29	162.57	< 0.001	0.31
Understanding eHealth Information	2.25 ± 14.52	6.48 ± 14.66	5.87 ± 14.68	60.96	< 0.001	0.40
Health behavior and decision-making changes		11.49 ± 19.70	11.86 ± 19.63	1.53	0.217	

SE: standard error

Regarding the information domain, computer/web knowledge was higher than that before the intervention (baseline: 8.47, post 1: 14.06, post 2: 12.94). A statistically significant difference was found in the score change over time (χ² = 60.04, *P* < .001). Regarding the motivation domain, perceived usefulness showed a tendency to slightly increase with a difference (baseline: 22.81, post 1: 24.61, post 2: 23.95), but there was no significant difference (χ² = 5.91, *P* = .052). The perceived ease of use was higher than pre-intervention and post-intervention (baseline: 20.12, post 1: 22.88), and slightly lower at post 2 (22.49); the result was statistically significant (χ² = 17.86, *P* < .001). Perceived enjoyment increased at post 1 (18.16) compared to baseline (15.66) and remained high at post 2 (17.41). This was confirmed to be statistically significant (χ² = 23.06, *P* < .001). Finally, the attitude toward eHealth information increased from 17.05 at baseline to 19.24 at post 1, and 18.83 at post 2, which was higher than that for pre-intervention; a significant change was noted (χ² = 18.47, *P* < .001). Regarding the behavioral skills domain, the eHealth literacy efficacy score was higher at post 1 (32.67) than at baseline (27.52). At post 2, the score remained high at 31.38, and there was a statistically significant difference (χ² = 72.76, *P* < .001).

Finally, the results of the variables related to the behavioral domain were as follows: both the searching performance score (χ² = 162.57, *P* < .001) and understanding score (χ² = 60.96, *P* < .001) were found to have significant differences between pre-and post-intervention. The searching performance scores increased significantly from 1.56 (baseline) to 23.06 (post 1) and maintained a significantly elevated score (9.65) at post 2. The understanding scores were as follows: baseline, 2.25; post 1, 6.48; and post 2, 5.87. The variables of health behavior and decision-making changes were measured twice, including post 1 and post 2, to assess the intervention’s effect on the participants’ health behavior change. The scores of health behavior and decision-making changes were confirmed to be 11.49 at post 1 and 11.86 at post 2 (range: 9–18). In addition, we confirmed the effect size of this program by comparing the correlation coefficient values before and after the intervention for each outcome variable. In the motivation domain, perceived ease of use (Cohen’s d: 0.88) and perceived enjoyment (Cohen’s d: 0.87) showed a large effect size of 0.8 or higher. Computer/web knowledge (Cohen’s d: 0.57) and attitude toward eHealth information Cohen’s d: 0.52) presented a medium effect size of ≥ 0.5, and a small effect size for the remaining outcome variables.

## Discussion

This study was conducted to explore an intervention program’s effects on improving eHealth literacy among older adults. This intervention was developed based on the IMB model, and its effectiveness was examined across all four areas of the IMB model. As a result, this intervention had a positive impact on changes in information, motivation, behavioral skills, and behaviors, related to eHealth information among older adults. The study’s main findings are as detailed below.

Firstly, the computer/web knowledge scores significantly increased in the information domain. The results of Xie’s two eHealth information-related intervention studies for older adults also showed an increase in general computer/web knowledge [24, 28]. The computer Internet-related knowledge part of this intervention was composed evenly from a relatively low level of knowledge to a high level of knowledge. Therefore, both beginners and intermediate participants reaffirmed the computer-related definitions and concepts vaguely known by them, which could lead to an increase in knowledge scores.

Secondly, in the motivation domain, perceived ease of use and perceived enjoyment significantly increased. Thus, it was easy and enjoyable for participants to search for health information on the Internet [39, 40]. The attitude toward eHealth information was significantly increased, implying that the participants regarded eHealth information as reliable, helpful, and accurate. However, perceived usefulness was found to be statistically insignificant, possibly because the participants had already recognized the usefulness of searching for health information on the Internet. The mean value of the perceived usefulness score was relatively high at baseline, suggesting that older adults were aware of the importance and necessity of using the Internet for health information, which eventually led to a desire for relevant education [41]. Currently, older adults generally do not have access to digital technology, and support systems are limited [42]. Therefore, to improve eHealth literacy, a media environment should be created that older adults can easily access [43] and continuously maintain their learning through the development and provision of programs at the government level [44, 45]. The participants were overwhelmingly positive about the intervention. Furthermore, they expressed their gratitude for the lessons they received on topics of interest.

Thirdly, eHealth literacy, which is a component of the behavioral skill domain, was statistically significant. According to the IMB model, information and motivation influence the behavioral skills. In other words, participants’ Internet-related knowledge becomes enriched [24, 28]. They find it easy and desirable to search for health information on the Internet [39, 40] and exhibit a positive attitude toward eHealth information [21], which improves their confidence in eHealth information searching methods and evaluations. In previous studies, the efficacy of older adults increased through interventions related to eHealth information [28]. Moreover, their digital efficacy increased in a study aimed at reducing the Internet gap [46]. In addition, discussion - a teaching channel of this intervention - could increase efficacy. The participants were able to promote efficacy by becoming peer trainers and providing positive feedback to each other through group activities such as discussions [46, 47].

Finally, in the behavior domain, the time required to search for health information on the Internet significantly shortened through the acquisition of Internet-related knowledge and repetitive training to access specific sites. However, the mean values ​​of health behavior and decision-making changes were measured only twice in the post 1 and post 2 evaluations, and were relatively not high. Two months might not be enough for a doctor’s visit or change certain health behaviors. According to a previous study, eHealth information and medical decision-making are significantly correlated [47].

### Strengths and limitations

This intervention was found to introduce positive changes in most outcome variables, possibly because of systematic and specific intervention contents by applying the IMB model [23] and the intervention mapping guidelines [27]. We used various teaching strategies and media and strengthened the learning effect by appropriately allocating lectures, practice, quizzes, and discussions in the intervention. In addition, to avoid relying on the instructor’s experience and teaching method, we applied a detailed and specific intervention protocol script. Thus, it was possible to maintain consistency in education among the four interventions.

However, this study has several limitations. Firstly, the participants’ level of education was generally high, which may have influenced their success in this study. The second limitation was the participants’ experience of using the Internet: of the total, 82.6% had already used the Internet, and 17 (37%) had used the Internet for more than five years. Therefore, participants with basic knowledge and experience related to the Internet could easily adapt to this intervention. In addition, we must consider the responsiveness to the research. During the five-week intervention, the researchers and participants formed a good relationship. This could improve the atmosphere of the class and positively affect learning. Paradoxically, such a relationship could influence the participants to modify their behavior according to the researcher’s expectations, which may threaten the validity of this study. Finally, the possibility that other internal factors may influence the results should be considered because this intervention was conducted without a control group.

## Conclusion

We found that the scores of 7 out of 9 variables derived from the IMB model increased statistically significantly through this intervention. Our findings suggest that the application of theories and methodologies can improve the quality of research in eHealth information literacy interventions. In addition, a variety of customized eHealth information education interventions should be developed, focusing on factors affecting the level of eHealth information acquisition in older adults (access to Internet media, Internet experience, education level, occupation, etc.) [48]. Specific measures should also be considered to increase the accessibility of interventions for older adults. Finally, we confirmed the effectiveness and feasibility of eHealth literacy intervention for older adults through this study. Therefore, we suggest a more highly controlled experimental design for future studies.

## Data Availability

The datasets generated and analyzed during the current study are not publicly available due personal information protection but are available from the corresponding author on reasonable request.

## References

[1] Rosenheck L (2008). Learning with ubiquitous computing. Educ Technol.

[2] International Telecommunication Union. 2020. Statistics. https://www.itu.int/en/ITU-D/Statistics/Pages/stat/default.aspx. Accessed 30 May 2021.

[3] Pew Research Center. 2021. Internet/broadband fact sheet. https://www.pewresearch.org/internet/fact-sheet/internet-broadband/ Accessed 30 May 2021.

[4] Ministry of Science and ICT & National Information Society Agency. 2019. Internet Usage Survey of Korea 2019. https://www.nia.or.kr/site/nia_kor/ex/bbs/View.do?cbIdx=99870&bcIdx=21930&parentSeq=21930. Accessed 30 May, 2021.

[5] Bujnowska-Fedak MM, Waligóra J, Mastalerz-Migas A (2019). The internet as a source of health information and services. Adv Innovat Health Sci.

[6] Turner AM, Osterhage KP, Taylor JO, Hartzler AL, Demiris G. A closer look at health information seeking by older adults and involved family and friends: design considerations for health information technologies. AMIA Ann Symp. Proc. 2018;2018:1036–45. PMC6371280 30815147

[7] Medlock S, Eslami S, Askari M, Arts DL, Sent D, De Rooij SE (2015). Health information–seeking behavior of seniors who use the internet: a survey. J Med Internet Res.

[8] Robertson-Lang L, Major S, Hemming H (2011). An exploration of search patterns and credibility issues among older adults seeking online health information. Can J Aging.

[9] Chung JH, Gassert CA, Kim HS (2011). Online health information use by participants in selected senior centres in Korea: current status of internet access and health information use by Korean older adults. Int J Older People Nurs.

[10] Weber W, Reinhardt A, Rossmann C (2020). Lifestyle segmentation to explain the online health information–seeking behavior of older adults: representative telephone survey. J Med Internet Res.

[11] Gazibara T, Kurtagic I, Kisic-Tepavcevic D, Nurkovic S, Kovacevic N, Gazibara T (2016). Computer and online health information literacy among Belgrade citizens aged 66–89 years. Health Promot Int.

[12] Seifert A, Schelling HR (2018). Seniors online: attitudes toward the internet and coping with everyday life. J Appl Gerontol.

[13] Bachl M (2017). Online health information seeking in Europe: do digital divides persist?. SCM Stud Commun Media.

[14] Cresci MK, Novak JM (2012). Information technologies as health management tools: Urban elders’ interest and ability in using the internet. Educ Gerontol.

[15] Chin J, Moeller DD, Johnson J, Duwe EA, Graumlich JF, Murray MD (2018). A multi-faceted approach to promote comprehension of online health information among older adults. Gerontologist.

[16] Freund O, Reychav I, McHaney R, Goland E, Azuri J (2017). The ability of older adults to use customized online medical databases to improve their health-related knowledge. Int J Med Inf.

[17] Xie B (2012). Improving older adults’ e-health literacy through computer training using NIH online resources. Libr Inf Sci Res.

[18] Chang SJ, Jang SJ, Lee H, Kim H (2020). Building on evidence to improve eHealth literacy in older adults: a systematic review. CIN: Comp Info Nurs.

[19] Heath G, Cooke R, Cameron E. A theory-based approach for developing interventions to change patient behaviours: a medication adherence example from paediatric secondary care. Healthcare. 3:1228–42. 10.3390/healthcare3041228. 10.3390/healthcare3041228 PMC4934641 27417822

[20] Watkins I, Xie B (2014). eHealth literacy interventions for older adults: a systematic review of the literature. J Med Internet Res.

[21] Chang SJ, Yang E, Lee K-E, Ryu H (2021). Internet health information education for older adults: a pilot study. Geriatr Nurs.

[22] Chang SJ, Choi S, Kim S-A, Song MS. Intervention strategies based on information-motivation-behavioral skills model for health behavior change: a systematic review. Asian Nurs Res. 2014;8:172 – 81. (2014), pp. 172–181. 10.1016/j.anr.2014.08.002.

[23] Fisher WA, Fisher JD, Harman J (2003). The information-motivation-behavioral skills model: a general social psychological approach to understanding and promoting health behavior. Soc Psychol Found Health Illn.

[24] Xie B (2011). Older adults, e-health literacy, and collaborative learning: an experimental study. J Ame Soc Inf Sci Technol.

[25] Kim YS, Tae YS, Jung K-I (2019). The development and evaluation of a health literacy-adapted self-management intervention for elderly cancer patients undergoing chemotherapy. J Kor Academy Nurs.

[26] Malone T, Jo P, Clifton S (2017). Perceived eHealth literacy and information behavior of older adults enrolled in a health information outreach program. J Consum Health Internet.

[27] Eldredge LKB, Markham CM, Ruiter RA, Fernández ME, Kok G, Parcel GS (2016). Planning health promotion programs: an intervention mapping approach.

[28] Xie B (2011). Effects of an eHealth literacy intervention for older adults. J Med Internet Res.

[29] Chang SJ, Im E-O (2014). A path analysis of Internet health information seeking behaviors among older adults. Geriatr Nurs.

[30] Venkatesh V, Bala H (2008). Technology acceptance model 3 and a research agenda on interventions. Decis Sci.

[31] Jung W-S, Kang H-G, Suk M-H, Kim E-H (2011). The use of the internet health information for the elderly. J Korean Public Health Nurs.

[32] Basu A, Dutta MJ (2008). The relationship between health information seeking and community participation: the roles of health information orientation and efficacy. J Health Commun.

[33] Norman CD, Skinner HA (2006). eHEALS: the eHealth literacy scale. J Med Internet Res.

[34] Chang SJ, Yang E, Ryu H, Kim HJ, Yoon JY (2018). Cross-cultural adaptation and validation of the ehealth literacy scale in Korea. Korean J Adult Nurs.

[35] Sharit J, Hernández MA, Czaja SJ, Pirolli P (2008). Investigating the roles of knowledge and cognitive abilities in older adult information seeking on the web. ACM Trans Comp-Hum Interact.

[36] Kaiser Family Foundation. 2005. e-Health and the elderly: how seniors use the Internet for health information. https://www.kff.org/wp-content/uploads/2013/01/e-health-and-the-elderly-how-seniors-use-the-internet-for-health-information-key-findings-from-a-national-survey-of-older-americans-survey-report.pdf. Accessed 30 May 2021.

[37] Fox S. Pew Research Center. 2006. Online health search 2006. https://www.pewresearch.org/internet/2006/10/29/online-health-search-2006/. Accessed 30 May 2021.

[38] Liu S, Dixon J, Qiu G, Tian Y, McCorkle R (2009). Using generalized estimating equations to analyze longitudinal data in nursing research. West J Nurs Res.

[39] Castilla D, Botella C, Miralles I, Bretón-López J, Dragomir-Davis AM, Zaragoza I (2018). Teaching digital literacy skills to the elderly using a social network with linear navigation: a case study in a rural area. Int J Human-Comput Stud.

[40] Xie B, Bugg JM (2009). Public library computer training for older adults to access high-quality Internet health information. Libr Inf Sci Res.

[41] Irizarry T, Shoemake J, Nilsen ML, Czaja S, Beach S, Dabbs A (2017). D. Patient portals as a tool for health care engagement: a mixed-method study of older adults with varying levels of health literacy and prior patient portal use. J Med Internet Res.

[42] Schreuers K, Quan-Haase A, Martin K (2017). Problematizing the digital literacy paradox in the context of older adults’ ICT use: aging, media discourse, and self-determination. Can J Comm.

[43] Levin-Zamir D, Bertschi I (2018). Media health literacy, eHealth literacy, and the role of the social environment in context. Int J Environ Res Public Health.

[44] Nymberg VM, Bolmsjö BB, Wolff M, Calling S, Gerward S, Sandberg M. ‘Having to learn this so late in our lives… Swedish elderly patients’ beliefs, experiences, attitudes and expectations of e-health in primary health care. Scand J Prim Health Care. 2019;37:41–52. https://doi.org/10.1080/02813432.2019.1570612. 10.1080/02813432.2019.1570612 PMC6452815 30732519

[45] Price-Haywood EG, Harden-Barrios J, Ulep R, Luo Q (2017). eHealth literacy: patient engagement in identifying strategies to encourage use of patient portals among older adults. Popul Health Manag.

[46] Gatti FM, Brivio E, Galimberti C (2017). “The future is ours too”: a training process to enable the learning perception and increase self-efficacy in the use of tablets in the elderly. Educ Gerontol.

[47] Chen Y-Y, Li C-M, Liang J-C, Tsai C-C (2018). Health information obtained from the internet and changes in medical decision making: questionnaire development and cross-sectional survey. J Med Internet Res.

[48] Arcury TA, Sandberg JC, Melius KP, Quandt SA, Leng X, Latulipe C (2020). Older adult internet use and eHealth literacy. J Appl Gerontol.

